# Inducible Defenses with a "Twist": *Daphnia barbata* Abandons Bilateral Symmetry in Response to an Ancient Predator

**DOI:** 10.1371/journal.pone.0148556

**Published:** 2016-02-17

**Authors:** Quirin Herzog, Max Rabus, Bernard Wolfschoon Ribeiro, Christian Laforsch

**Affiliations:** 1 Department of Biology II, Ludwig-Maximilians-University Munich, Planegg-Martinsried, Germany; 2 Department of Animal Ecology I, University of Bayreuth, Bayreuth, Germany; 3 BayCEER, University of Bayreuth, Bayreuth, Germany; Evolutionary Biology Centre (EBC), Uppsala University, SWEDEN

## Abstract

Predation is one of the most important drivers of natural selection. In consequence a huge variety of anti-predator defenses have evolved in prey species. Under unpredictable and temporally variable predation pressure, the evolution of phenotypically plastic defensive traits is favored. These “inducible defenses”, range from changes in behavior, life history, physiology to morphology and can be found in almost all taxa from bacteria to vertebrates. An important group of model organisms in ecological, evolutionary and environmental research, water fleas of the genus *Daphnia* (Crustacea: Cladocera), are well known for their ability to respond to predators with an enormous variety of inducible morphological defenses. Here we report on the “twist”, a body torsion, as a so far unrecognized inducible morphological defense in *Daphnia*, expressed by *Daphnia barbata* exposed to the predatory tadpole shrimp *Triops cancriformis*. This defense is realized by a twisted carapace with the helmet and the tail spine deviating from the body axis into opposing directions, resulting in a complete abolishment of bilateral symmetry. The twisted morphotype should considerably interfere with the feeding apparatus of the predator, contributing to the effectiveness of the array of defensive traits in *D*. *barbata*. As such this study does not only describe a completely novel inducible defense in the genus *Daphnia* but also presents the first report of a free living Bilateria to flexibly respond to predation risk by abandoning bilateral symmetry.

## Introduction

Phenotypically plastic defensive traits in prey organisms typically evolve in environments characterized by strong variation in predation risk. These traits, termed inducible defenses, are known to affect organismic interactions and population dynamics and are therefore crucial for the understanding of ecosystem functioning and evolutionary processes (e.g. [1,2]). The prerequisites for inducible defenses to evolve include, next to the variable and unpredictable predation risk, the existence of a cue that reliably indicates the presence of the predator, the effectiveness of the defense and finally defense-associated costs, which exceed the benefits in the absence of the threat [3]. Inducible defenses can be expressed on the level of behavior, life history, physiology and morphology and are found in almost all taxa ranging from bacteria and unicellular organisms to vertebrates (e.g. [4,5]).

Water fleas of the genus *Daphnia* (Crustacea: Cladocera) are important model organisms in ecological, evolutionary and environmental research. They are well known for their ability to respond to predators with an enormous variety of inducible morphological defenses, which are thought to function by impeding handling and ingestion by the predator [6]. So far primarily helmet-like (e.g. enlarged and pointy helmets, dorsal crests), spine-like (e.g. elongated tail spines, neckteeth) or structural (i.e. fortification of the carapace) defenses have been reported from this genus [7]. With the exception of the development of a spiky helmet and a longer tail spine in *D*. *lumholtzi* exposed to fish [8], most of these inducible morphological defenses are expressed in response to predatory insects, e.g. phantom midge larvae and back swimmers, and pelagic carnivorous crustaceans, e.g. cyclopoid copepods and *Leptodora*. Over the last years, another crustacean predator, the pond dwelling tadpole shrimp *T*. *cancriformis*, and responses of its prey received increasing attention. Being extant for 220 million years, this most ancient animal species acts as strong selective force on coexisting *Daphnia* species. Intriguing morphological defenses such as a “crown of thorns” in the *D*. *atkinsoni* species complex [9] and the “bulkiness” in *D*. *magna* [10] are attributed as effective means against *Triops* predation. Recently, it has been shown that the African species *D*. *barbata*, which coexists with *Triops* in temporary freshwater ponds and lakes, responds to *T*. *cancriformis* and the backswimmer *Notonecta glauca* with specialized defenses which are based on the same structures (e.g. helmet, tail spine, dorsal ridge), but built in a different shape [11]. Both induced defenses have been shown to enhance survival when the daphnids are exposed to the respective predator. Based on the latter study, we here report on a unique inducible morphological defense in *D*. *barbata*, the body torsion.

## Results and Discussion

**Fig 1:**
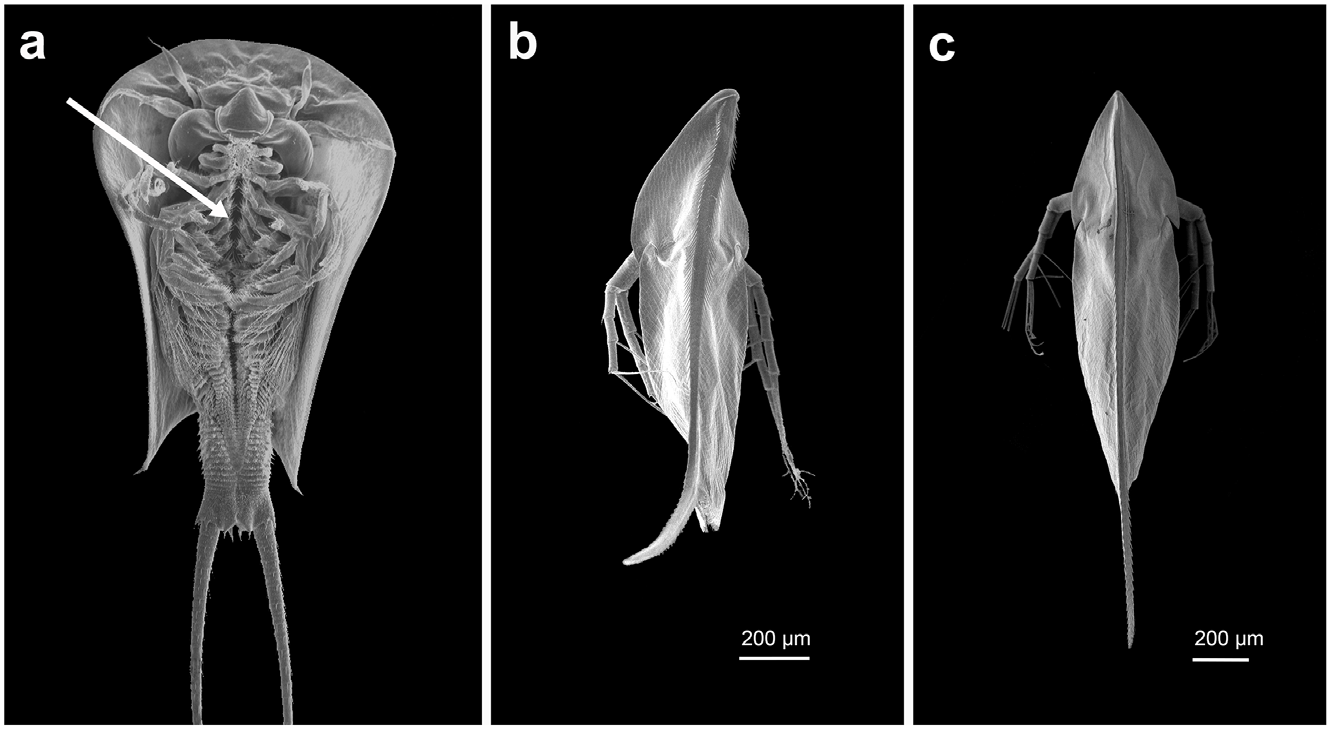
Scanning electron microscope images of the experimental animals. a) *Triops cancriformis*, ventral view with the arrow pinpointing to the narrow food groove. The ancient predator feeds on *Daphnia*, which are caught, subsequently placed into the food groove and transported towards the mandibles; b) Dorsal view of a *Triops*-exposed morph of *Daphnia barbata* showing the “twisted” appearance. The tips of helmet and tail spine deviate from the body axis in opposite directions, leading to an S-shaped dorsal ridge and thus abolishing bilateral symmetry of the individual. The twisted morphotype can be assumed to severely impede the transport through the food groove as it should cause the daphnid to wedge within the food groove of the predator. c) Dorsal view of *D*. *barbata* not exposed to the predator. The dorsal ridge aligns with the bilateral body axis, the tips of helmet and tail spine do not deviate from the body axis.

**Fig 2:**
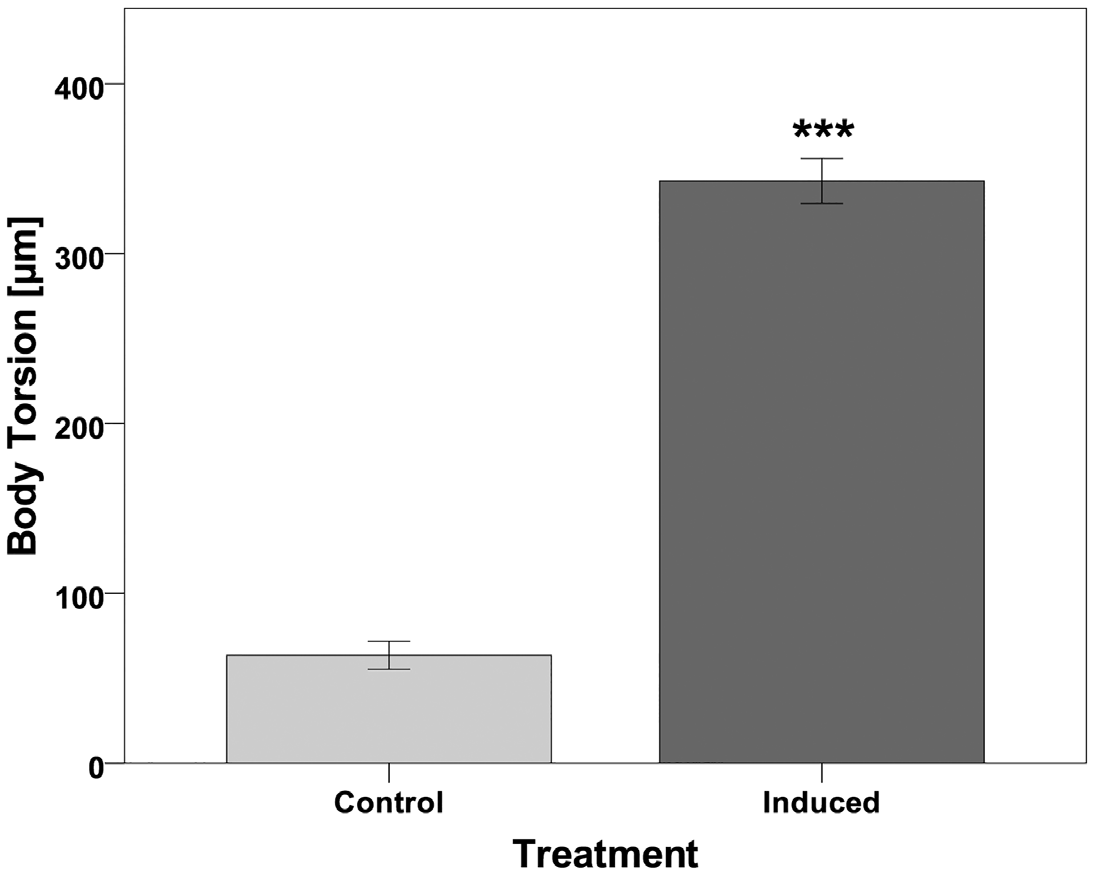
Comparison of the body torsion in non-predator-exposed (Control) and predator-exposed (Induced) primiparous *Daphnia barbata*. Body torsion is here defined as the sum of helmet and tail spine deviation from the body axis. The error bars show the standard error of Mean (SE), the asterisks indicate the significance level (*** *P* < 0.001) based on a F-Test (*F*(1, 22) = 264.09).

*Triops*-exposed *D*. *barbata* alter their body symmetry in response to this predator (Fig 1). In detail, this change is characterized by a torsion of the whole body that leads to an S-shaped dorsal ridge. As both tail-spine and helmet are bent backwards the torsion further results in both structures to point into opposite directions as they laterally deviate from the body axis. Interestingly, the orientation of this change is apparently not random, but genetically fixed: All measured specimens had their helmets pointed to the right and the tail spine to the left from a dorsal view. As a consequence, the bilateral axis, which normally aligns along the dorsal ridge in *D*. *barbata*, is abolished. The body torsion, here quantified as the sum of helmet- and tail spine deviation from the body axis (defined as the line connecting the base of the tail spine and the middle between the fornices of the shoulder shield), was significantly increased in predator-exposed daphnids compared to the control morph not exposed to predator released cues (control: 63.47μm ± 25.97 SD; predator-exposed: 342,87μm ± 49,51 SD; F-Test, *F*(1, 22) = 264,09, *P* < 0.001; Fig 2). Therefore, the twisted body can be considered to be predator-induced.

The body torsion likely contributes considerably to the increased survival rate of *Triops*-induced *D*. *barbata*, as it should act synergistically with or additively to the previously described induced traits, i.e. the elongated, dorsally bent helmet, the curved and dorsally bent tail spine, and more and larger spinules on the dorsal ridge [11]. The functioning of the body torsion may be explained by the way *Triops* is handling its prey [12]: the prey is caught by encaging it with the numerous legs and placed into the narrow food groove, a symmetrical, conveyor-like structure built by the gnathobases of the trunk limbs. Then it is transported towards the mandibles through movements of the limbs. When *Triops* catches a *Daphnia*, it is almost exclusively placed in the food groove in a way that the dorsal side of the prey faces the predator while the head of the daphnid points towards the mandibles (Rabus, unpublished observation). Given this mode of feeding, we expect the body torsion to effectively interrupt the transport within the food groove. The sidewards bent helmet and the sidewards curved tail spine may each thread into opposing small gaps between the gnathobases, causing the daphnid to become wedged within the food groove. This would in turn cause a complete blockage of the transport of the daphnid towards the mandibles, which should require *Triops* to release its prey from the food groove in order to rearrange its position, giving the daphnid the chance to escape.

In contrast to the classical defensive traits in *Daphnia*, e.g. spines or helmets, the observed body torsion in *D*. *barbata* causes a massive change in the morphology of the whole body since not only the helmet and the tail spine deviate from the body axis, but also the carapace is twisted. This morphological alteration is so far unique since no other free living animal has been shown to completely abandon its bilateral symmetry as an induced response to predation. So far, only few ontogenetically determined deviations from bilateral symmetry have been described [13]. Only two cases of predator-induced asymmetry are known at this point: a one-sided enlargement of a single spine in the rotifer *Keratella tropica* exposed to the predatory rotifer *Asplanchna* [14]; and the sessile barnacle *Chthamalus anisopoma*, which changes its shell shape from the typical conical morph to an atypical “bent-over” morph when exposed to the carnivorous gastropod *Acanthina angelica*, resulting in a shift of the bilateral axis [15]. In *D*. *barbata* however, the bilateral axis is not changed to another plane, but abolished completely, leaving the animal without a symmetrical axis. This tremendous change in morphology should considerably alter the hydrodynamic properties of the induced individuals. This may negatively affect locomotion leading to ecological costs (e.g. escape behavior against other predators) and physiological costs, respectively. Additionally, the twisted carapace may also affect feeding efficiency, i.e. by impairing the suction-and-pressure pump built by the thoracic limbs and the carapace [16], and possibly also reproduction, i.e. by limiting the available space in the brood pouch. Finally, the formation of the body torsion itself likely incurs developmental costs. In sum, this suggests high costs associated with the body torsion. Since it is an evolutionary prerequisite that an inducible defense provides a net benefit under predation [17], this is an indication that the body torsion plays an essential role in the defense against the predator *Triops*. It is therefore likely that the previously described increase in survival rate [11] is to a great extent caused by this trait.

To conclude, we report on the “twist”, a torsion of the whole body, as an intriguing novel inducible defense in the extensively studied model genus *Daphnia*. Hence, our finding further adds to the emerging awareness of the complexity of inducible morphological defenses in *Daphnia*, which often include a full array of morphological alterations, ranging from prominent structures (e.g. helmets and spines) to minute (e.g. the tiny spinules along the dorsal ridge) or even “hidden” defenses (e.g. a fortified exoskeleton). Therefore, it also shows the need for further studies to reveal and entangle the mechanisms underlying the effectiveness of these defenses. Moreover, body torsion presumably requires a complex developmental pattern for its formation. Since the symmetry of adult Bilateria is usually established during the cleavage period [18], the deviation from this symmetry in later life stages must apparently be triggered by well-defined interactions of genes with the environment. Given that *Daphnia* has emerged as important model organism for biomedical research and environmental genomics [19,20], the body torsion in *D*. *barbata* might be an extraordinary model system for understanding the developmental mechanisms underlying phenotypic variations.

## Material and Methods

The specimens of *D*. *barbata* analyzed in this study derive from the induction experiment described in detail in Herzog & Laforsch [11]. In this study, we used a single clone of *D*. *barbata* (Eth 1), originating from Ethiopia and a laboratory cultured clonal line of *T*. *cancriformis* as predator. The induction experiment was conducted in a temperature controlled climate chamber at 20 ± 0.5°C under fluorescent light with a constant photoperiod (15h light: 9h dark). As starting point for the induction experiment, two stable cultures (control and *Triops*-induced) were established starting with 20 adult, brood bearing *D*. *barbata* in 1.5L glass beakers containing semi artificial medium based on ultrapure water, well water, phosphate buffer and trace elements. A net cage (mesh width: 125μm) was placed in each beaker and was either empty (control) or stocked with a single *T*. *cancriformis* (*Triops*-induced). The daphnids were fed daily with 1 mg C/L of the green alga *Scenedesmus obliquus*, *Triops* were fed daily with 5 to 10 live *D*. *barbata* and 3 live red chironomid larvae. Every 5 days half of the medium was exchanged. Every week, randomly sampled juveniles, less than 2 days old, were transferred from the starting cultures into new beakers, each representing a biological replicate, which were treated as described above. As soon as the daphnids in the new beakers reached primiparity, they were preserved in 70% ethanol and stored until further analysis.

To quantify the body torsion, we measured the deviation of the tip of the helmet and the tip of the tail spine from the body axis. Since the body axis becomes asymmetric in predator exposed animals, it is here defined as the line connecting the base of the tail spine and the middle between the fornices of the shoulder shield. Then the torsion was calculated as the sum of helmet and tail spine deviation from the body axis. Mean torsion was calculated for each replicate (control N = 10; induced N = 14), as several individuals (on average 4) from each replicate were measured (S1 Dataset). Then the data was tested for normality and homogeneity of variance and a F-Test was conducted to test for treatment-dependent differences in body torsion.

## Supporting Information

S1 Dataset Body Torsion in *D*. *barbata*, including replicate means. (XLSX)
